# The Outcome of Repeated Mid Urethral Sling in SUI Treatment after Vaginal Excisions of Primary Failed Sling: Preliminary Study

**DOI:** 10.1155/2016/1242061

**Published:** 2016-11-23

**Authors:** Jacek Kociszewski, Wojciech Majkusiak, Andrzej Pomian, Paweł Tomasik, Edyta Horosz, Andrzej Kuszka, Ewa Barcz

**Affiliations:** ^1^Evangelisches Krankenhaus Hagen-Haspe, Hagen, Germany; ^2^1st Department of Obstetrics and Gynecology, Medical University of Warsaw, 1/3 Starynkiewicza Sq., 02-015 Warsaw, Poland

## Abstract

Mid urethral sling is the standard in SUI treatment. Nevertheless, the risk of reoperation reaches 9%. There is no consensus as to the best treatment option for complications. A question is raised: what is the optimal way to achieve the best result in patients after primary failure? The aim of the study was to evaluate the outcomes of repeat MUS surgery in patients after excision of the sling with recurrent SUI. We compared its effectiveness with uncomplicated cases treated with TVT. 27 patients who underwent the repeated MUS and 50 consecutive patients after primary TVT were enrolled in the study. After 6 months, we have found that 24 (88.46%) patients from repeat sling group and 48 (96%) patients after primary sling were dry (1-hour pad test, 2 g or less). The difference between groups was not significant. We showed statistically significant improvement of quality of life in both groups. In conclusion, we showed that repeated sling after MUS excision is almost as effective as primary MUS. We postulate that sling excision and repeated MUS may be the best option for persistent SUI and/or complications after MUS procedures. Further multicenter observations are ongoing as to provide results on bigger group of cases.

## 1. Introduction

Mid urethral sling is the gold standard in stress urinary incontinence treatment [1]. The effectiveness of the procedure is estimated for 70–95%; nevertheless, the risk of reoperation reaches even 9% [2]. Indications for reoperations are usually lack of effectiveness, voiding dysfunctions, OAB de novo, pain, or recurrent infections [3].

There is no consensus as to the best treatment option for complications. In the armamentarium, there is implantation of second sling without removal of the first one in case of failure, sling incision in case of voiding dysfunctions [4], and vaginal excision of the sling in case of voiding dysfunction, OAB de novo, pain, and so forth [5]. A question of major importance is raised: what is the optimal way to achieve resolving of complications and at the same time to achieve the best result in SUI treatment in patients with primary failure?

The vaginal excision of the sling is a safe procedure that resolves most of complications such as voiding dysfunctions, OAB de novo, and pain; nevertheless, in over 60% of patients SUI recurrence is present [5].

The aim of the present study was to evaluate the outcomes of repeat MUS surgery in patients after excision of vaginal portion of the failed sling with persistent or recurrent SUI. We compared the effectiveness of the repeated sling procedure with uncomplicated cases of pure stress incontinences treated with TVT. The outcome of the surgery was evaluated as objective and subjective cure rate after at least six months after the surgery.

## 2. Materials and Methods

27 patients who underwent the repeated MUS implantation after first sling vaginal excision in 1st Department of Obstetrics and Gynecology of Medical University of Warsaw between 2013 and 2015 were enrolled in the study.

The excisions of the first sling were performed mainly because of the failure of the surgery (85%) and in other cases because of other complications (32%) (such as OAB or urinary retention). Some of the patients suffered from more than one symptom (i.e., persistent SUI and OAB). All the patients who underwent repeated sling implantation presented pure stress urinary incontinence without other symptoms (they were resolved by first sling excision). The degree of SUI after first surgery (first sling implantation) before its excision was determined using 1-hour pad test, cough test, and IIQ7 scoring. The time of repeated tape implantation after excision of the failed one was from 80 to 100 days.

One surgeon using the same technique performed the sling excisions. After localization of the tape in ultrasound examination, the vagina was incised beneath the sling. The Hegar maneuver was used to facilitate the tape preparation. Then the sling was grasped with two Peans and incised beneath the urethra. The arms of the sling were then prepared from the surrounding tissues in their vaginal part and then excised.

In control group, we analyzed 50 consecutive patients after the TVT implantation from 1 January to 30 June in 2015.

Preoperative assessment in both groups consisted of detailed medical and surgical history, urogynecologic examination, a 1-hour pad test, cough stress test, urine analysis, multichannel urodynamic evaluation, and pelvic floor ultrasonography [6] and additionally all patients completed the Incontinence Impact Questionnaire (IIQ7).

Patients after sling excision were operated by only one surgeon using the standard TVT procedure (Gynecare; Ethicon Inc., Somerville, NJ). In all cases, surgical procedures were performed as previously described according to 1/3 rule after PF ultrasound evaluation of urethra length [7]. Tensioning of the tapes for TVT was achieved by cough test. 3 trained gynecologists performed surgeries in control group.

All patients were followed up at 1 day, 1 month, 6 months postoperatively, and every second year thereafter. Pelvic floor ultrasound, 1-hour pad test, cough test, IIQ7, and pelvic examinations were completed within 6 months and every second year of follow-up.

The tape location after sling implantation was assessed using pelvic floor ultrasound examination as it was described previously [7]. In summary, the distance from proximal edge of the tape to the echolucent urethral lumen and the distance from the middle part of the tape to the bladder neck were measured.

Statistical analysis was performed using Chi-square and Mann–Whitney* U* tests.

## 3. Results

Between 2013 and 2015, 116 patients with complications after MUS procedure were diagnosed in the department. 71 of them underwent vaginal sling removal, and 40 underwent repeated sling implantation. Till now, 27 patients (62.4 ± 8.4 years) completed 6-month observation period and were included in the study. Patients after third or fourth sling procedure as well as after bladder or urethral injury were excluded from the study.

In the group of patients with complications, the main reason of sling removal was persistent SUI (14 patients), OAB de novo after the surgery (1 patient), and persistent SUI with OAB (12 patients). In the group with unsuccessful treatment in general sling was located in proximal part of urethra.

In 16 cases (59.3%), a primary sling was TOT, there was a retropubic sling in 4 cases (14.8%), and in 2 cases there were mini slings (7.4%). In 5 patients, we did not obtain information about first procedure.

The average time between primary sling excision and repeat TVT was 3 months.

Mean age of patients from control group was 58.3 ± 9.4.

In Table 1, the results of 1-hour pad test and IIQ7 in analyzed patients after first sling excision and from control group (initial examination before TVT implantation) were summarized. The differences between two groups in 1-hour pad test and IIQ7 score were not statistically significant.

After 6 months of observation, we have found that 24 (88.46%) patients from the group after repeated sling group and 48 (96%) patients after primary sling implantation were dry (1-hour pad test, 2 g or less). The difference in percentage of negative pad test after primary and repeated procedure was not statistically significant. There were statistically significant differences (*p* < 0.01) in IIQ7 score in analyzed groups.

In Table 2, results in 1-hour pad test and IIQ7 score performed after 6 months after repeated sling implantation and primary sling implantation were summarized.

In successfully treated patients after repeated sling (*n* = 24), IIQ7 score did not defer significantly from the control group's results.

Analysis of failure of the TVT surgery showed that in 5 cases of unsuccessfully treated patients in both groups the sling was implanted in proximal part of urethra.

In patients who remained dry 6 months after the surgery, the location of the sling was in distal 1/3 part of urethra, approximately 3.3 mm from echolucent urethral lumen in ultrasound examination (Table 3).

## 4. Discussion

The primary aim of the study was to evaluate the results of repeated MUS procedure after vaginal sling excision in unsuccessfully or complicated patients with SUI.

The most common reason for sling excision was persistent incontinence and in half cases persistent incontinence with concomitant OAB de novo. In patients treated with repeated sling, we showed that the objective cure (negative cough and 1-hour pad test) was achieved in 89% of patients after repeated sling as compared to 96% in primary sling cases. The difference between repeated sling and primary sling in our group was not statistically significant.

We also obtained great improvement in quality of life (measured as IIQ7 score) in both groups; nevertheless, the improvement was more pronounced in primary sling patients.

Nowadays, there is a worldwide discussion regarding the best way of unsatisfactory results after MUS procedures as there are a lot of treatment options.

The first step in the attempt to provide the best way of treatment for the complicated patients is the proper diagnosis of failure and indications for sling revision.

In analysis of Unger et al., the main purposes for sling revision (incision or partial or complete excision) were urinary retention, LUTS, and recurrent infections. The percentage of LUTS was similar to that in our group; nevertheless, the authors did not analyze cases of persistent SUI in their center [8]. In complicated cases, another important reason for sling excision is the sling exposure that results in recurrence of SUI [9].

In case of LUTS (urinary retention, OAB, and voiding difficulties), different treatment approaches are discussed.

One option is sling release. The technique of releasing differs according to the time in which complications are diagnosed. In Rautenberg et al.'s analysis, authors showed that early tape mobilization provides resolution of symptoms in almost all patients [10].

What is important is that such procedure is possible during first week after the sling implantation. In case of later diagnosis, there is no possibility of sling mobilization. In such case, the sling release (incision or partial or complete excision) must be taken into consideration. Sling incision is one of the options. In analysis of 100 cases from Mayo Clinic, global improvement and satisfaction were reported by 41% of patients after the procedure [11]. The main purpose of low satisfaction after sling incision is the recurrence of SUI which occurs in over 60% of patients after sling incision [4]. Another approach is complete sling excision that is even more accurate in LUTS resolution but naturally causes recurrent SUI in similar percentage of cases as after sling incision [5].

According to literature data and our experience, complete sling excision in case of urinary retention, OAB, sling exposure, infections, and so forth is the most effective procedure as far as the resolution of above complications is concerned.

On the other hand, there is a group of patients with persistent SUI after MUS procedure. Therapeutic option for them is second sling implantation, sling shortening, and repeated sling after sling excision. Meyer et al. showed 77% of successfully treated patients after second sling in the group of patients with persistent SUI with persistent urethral hypermobility [12]. In analysis of subjective cure rate after repeated sling, the significant lower satisfaction was shown after second sling as compared to the primary sling implanted (62% versus 86%) [13]. In another observation, cure rate after repeated sling reached 79% [14]. In case of persistent SUI, the tape shortening is another option for patients. In comparison of cure rates in patients after repeated sling and after sling shortening, it was shown that repeated sling was much more effective than the other option (72% versus 46%) [15].

In many cases of unsuccessful treatment of SUI, we have to deal with complex problems such as persistent SUI with OAB, retention, pain, or sling exposure. Taking the above into account, we should choose the option that allows obtaining the complications resolution and on the other hand provides the best conditions for secondary treatment. As it was previously shown, the complete sling excision is more efficient in OAB and pain treatment; we would like to show the effectiveness of the repeated sling after sling excision as well as the results of that procedure in cases with persistent SUI.

We showed that repeated sling after MUS excision had the same effectiveness (as far as the continence is considered in objective tests: negative cough test and negative 1-hour pad test) as the primary implanted sling (89 versus 96%; ns). As far as the subjective cure rate is considered, the results were slightly worse than those after the first sling, something that was connected mainly with emotional status of the patients (anxiety and depression). What is worth mentioning is that we observed lower IIQ7 score after 2 years of observation (as patients reported that they started to believe in success).

The main limitation of the study is small patients group. It also should be stressed that not in all cases of failed sling the repeated one will be the best option because the most important inclusion criterion was pure stress incontinence after first sling excision.

In conclusion, we showed that repeated sling after MUS complete excision is probably almost as effective as primary MUS. We postulate that vaginal sling excision and repeated MUS may be the best option for persistent SUI and/or complications after mid urethral sling procedures. Further multicenter observations are ongoing to provide the result based on bigger group of cases.

## Figures and Tables

**Table 1:** Results of 1-hour pad test and IIQ7 in the group of patients after sling excision and in control group.

	1-hour pad test [g]		IIQ7 score [0–100]	
	After sling excision	Control group before TVT	After sling excision	Control group before TVT
Mean	121.52	81.14	85.90	77.54
Median	115	44.50	85.71	76.19
Std. dev.	65.87	97.84	12.88	16.21

**Table 2:** Results in 1-hour pad test and IIQ7 score performed 6 months after repeated sling implantation and primary sling implantation.

	1-hour pad test [g]		IIQ7 score [0–100]	
	Repeated MUS	Primary MUS	Repeated MUS	Primary MUS
*N*	27	50	27	50
Mean	10.92	0.92	19.05	4.10
Median	0	0	4.76	0
Std. dev.	41.32	5.26	30.21	11.18

**Table 3:** Tape location 6 months after repeated sling implantation.

(*N* = 24)	Distance from the bladder neck		Distance from the echolucent urethral lumen
	[mm]	% of urethral length	[mm]
Mean	19.43	64.9%	3.13
Median	19.00	64.5%	3.30
Std. dev.	3.19	6.9%	0.94

## References

[1] Ford A. A., Rogerson L., Cody J. D., Ogah J. (2015). Mid-urethral sling operations for stress urinary incontinence in women. *The Cochrane Database of Systematic Reviews*.

[2] Fialkow M., Symons R. G., Flum D. (2008). Reoperation for urinary incontinence. *American Journal of Obstetrics and Gynecology*.

[3] Kociszewski J., Kolben S., Barski D., Viereck V., Barcz E. (2015). Complications following tension-free vaginal tapes: accurate diagnosis and complications management. *BioMed Research International*.

[4] Viereck V., Rautenberg O., Kociszewski J., Grothey S., Welter J., Eberhard J. (2013). Midurethral sling incision: Indications and outcomes. *International Urogynecology Journal and Pelvic Floor Dysfunction*.

[5] Fabian G., Kociszewski J., Kuszka A. (2015). Vaginal excision of the sub-urethral sling: analysis of indications, safety and outcome. *Archives of Medical Science*.

[6] Tunn R., Albrich S., Beilecke K. (2014). Interdisciplinary S2k guideline: sonography in urogynecology. *Geburtshilfe und Frauenheilkunde*.

[7] Kociszewski J., Rautenberg O., Kuszka A., Eberhard J., Hilgers R., Viereck V. (2012). Can we place tension-free vaginal tape where it should be? the one-third rule. *Ultrasound in Obstetrics and Gynecology*.

[8] Unger C. A., Rizzo A. E., Ridgeway B. (2016). Indications and risk factors for midurethral sling revision. *International Urogynecology Journal*.

[9] Linder B. J., El-Nashar S. A., Carranza Leon D. A., Trabuco E. C. (2016). Predictors of vaginal mesh exposure after midurethral sling placement: a case–control study. *International Urogynecology Journal and Pelvic Floor Dysfunction*.

[10] Rautenberg O., Kociszewski J., Welter J., Kuszka A., Eberhard J., Viereck V. (2014). Ultrasound and early tape mobilization—a practical solution for treating postoperative voiding dysfunction. *Neurourology and Urodynamics*.

[11] Kim-Fine S., El-Nashar S. A., Linder B. J. (2016). Patient satisfaction after sling revision for voiding dysfunction after sling placement. *Female Pelvic Medicine & Reconstructive Surgery*.

[12] Meyer F., Hermieu J. F., Boyd A. (2013). Repeat mid-urethral sling for recurrent female stress urinary incontinence. *International Urogynecology Journal and Pelvic Floor Dysfunction*.

[13] Stav K., Dwyer P. L., Rosamilia A. (2010). Repeat synthetic mid urethral sling procedure for women with recurrent stress urinary incontinence. *The Journal of Urology*.

[14] Lee K.-S., Doo C. K., Han D. H., Jung B. J., Han J.-Y., Choo M.-S. (2007). Outcomes following repeat mid urethral synthetic sling after failure of the initial sling procedure: rediscovery of the tension-free vaginal tape procedure. *Journal of Urology*.

[15] Han J.-Y., Moon K. H., Park C. M., Choo M.-S. (2012). Management of recurrent stress urinary incontinence after failed midurethral sling: tape tightening or repeat sling?. *International Urogynecology Journal*.

